# The use of Local Ecological Knowledge as a complementary approach to understand the temporal and spatial patterns of fishery resources distribution

**DOI:** 10.1186/s13002-017-0156-9

**Published:** 2017-06-01

**Authors:** Mauro Sergio Pinheiro LIMA, Jorge Eduardo LINS OLIVEIRA, Marcelo Francisco de NÓBREGA, Priscila Fabiana Macedo LOPES

**Affiliations:** 1 Department of Oceanography and Limnology at the Federal University of Rio Grande do Norte, Natal, Brazil; 2 Fishing Ecology, Management and Economics group, Department of Ecology at the Federal University of Rio Grande do Norte, Natal, RN Brazil

**Keywords:** Artisanal fishing, Fisheries management, Local ecological knowledge, Northeast Brazil, *Caranx* crysos, *Scomberomorus brasiliensis*, *Lutjanus synagris*

## Abstract

**Background:**

Acquiring fast and accurate information on ecological patterns of fishery resources is a basic first step for their management. However, some countries may lack the technical and/or the financial means to undergo traditional scientific samplings to get such information; therefore affordable and reliable alternatives need to be sought.

**Methods:**

We compared two different approaches to identify occurrence patterns and catch for three main fish species caught with bottom-set gillnets used by artisanal fishers from northeast Brazil: (1) scientific on-board record data of small-scale fleet (*n* = 72 trips), and (2) interviews with small-scale fishers on Local Ecological Knowledge (LEK) (*n* = 32 interviews). We correlated (Pearson correlations) the months cited by fishers (LEK) as belonging to the rainy or to the dry season with observed periods of higher and lower precipitation (SK). The presence of the three main fish species at different depths was compared between LEK and SK by Spearman correlations. Spearman correlations were also used to compare the depths of greatest abundance (with the highest Capture per Unit Effort - CPUE) of these species; the CPUEs were descendly ordered.

**Results:**

Both methods provided similar and complementary bathymetric patterns of species occurrence and catch. The largest catches occured in deeper areas, which also happened to be less intensively fished. The preference for fishing in shallower and less productive areas was mostly due to environmental factors, such as weaker currents and less drifting algae at such depths.

**Conclusion:**

Both on-board and interview methods were accurate and brought complementary information, even though fishers provided faster data when compared to scientific on-board observations. When time and funding are not limited, integrative approaches such as the one presented here are likely the best option to obtain information, otherwise fishers’ LEK could be a better choice for when a compromise between speed, reliability and cost needs to be reached.

**Electronic supplementary material:**

The online version of this article (doi:10.1186/s13002-017-0156-9) contains supplementary material, which is available to authorized users.

## Background

The efficient management of fishery resources minimally involves the understanding of seasonality and species distribution [1, 2]. These factors are crucial to the dynamics of small and large-scale fishing, as they are known to influence the life cycle, abundance, biomass and species richness [3–5], and for determining spawning [6] and food aggregations [6, 7].

Density dependent and independent factors limit the spatial distribution of populations and species, therefore influencing individual survival and reproduction, and population abundance [8]. Understanding how fishing and environmental variability interact to produce an effect on exploited populations (target species) has been an evolving and intriguing question in fisheries science for decades [9], being a limiting factor to the appropriate management of fishing stocks. Adding to that, there is also the question that fisheries have to be evaluated in the context of a changing environment [10–12], which is better done under an ecosystem approach [13–15]. However, an ecosystem approach requires the integration of the spatial dynamics and the seasonal variability of the various components of the fishery, which include not only the natural resources, but also the fishers [10]. Fishers retain specific knowledge of fishing resources that could be crucial to support an ecosystem approach to fisheries (EAF) in developing tropical countries, because these are places where there are often poor or no data on the status of fish stocks at local or regional scales [16, 17].

In EAF, one of the first decisions to be made regards the need to establish fishing boundaries, which can be done through spatial jurisdictions, regional fisheries institutions or natural physical and technological (limits imposed by the fleet autonomy, for instance) boundaries. The scale of a fishery system can vary greatly and ecosystems are not always clearly defined entities with unambiguous boundaries. Human dimensions, with identification and involvement of stakeholders, are central to EAF. Understanding their values, needs, aspirations, and current livelihood circumstances is key to informing policy and influencing management decisions [18]. It is also important to identify the scale of the fishery, as industrial and small-scale fisheries are completely different realms, with distinct fish targets, dynamics (intensity, gear, depth, and season) and markets.

For the small scale fisheries, the understanding of the environmental factors that affect and determine their catches over temporal and spatial scale is usually limited and often inferred without sound scientific information or technological geolocation support [19]. Their industrial counterparts rely on scientific and technological advances that identify seasonal and bathymetric patterns of fishery resources, allowing them to optimize efforts to maximize catches from the continental shelf to abyssal zones [20].

Small-scale fisheries are not restricted to the tropics or the developed countries, but these are the regions where most of these fisheries exist [21]. The tropical seasonality is defined by marked rainy and dry periods that influence the life cycle, and therefore, the spatial-temporal occurrence of many commercial species [22, 23]. Small-scale fisheries have their traditional grounds, in accordance to the specific periods of aggregations and migrations of fish stocks. For example, in the Brazilian northeast, summers (dry season) are the period to catch groupers and snappers at the continental shelf edge [24], whereas the serra Spanish mackerel is caught in the rainy months all over the northern coast of South America [25].

Such need to perceive the environment through its natural clues is likely to explain why fishers have an understanding that is not limited to the recognition of the spatiotemporal patterns of their target species [1]. Fishers have enough accumulated knowledge to make them sensitive to some environmental changes, with the ability to interpret them, and provide production estimates [26]. Such fishers’ ecological knowledge (LEK) is especially important in areas with scarce information on fishing statistics, and it can be sometimes the only information available to build up fisheries management strategies [17].

Traditionally fisheries management recommendations tended to demand complex research models and large amounts of statistical data based only on conventional scientific information, which, despite all the time, funding and expertise involved, may also provide controversial estimates [27–29]. Such limitations, exacerbated in countries where science and statistics are not a priority, brought to the forefront the need to look beyond the scientific paradigm, and learn how to access information that is affordable and quickly available [30, 31].

Fishers’ LEK has been proposed as such a solution to restore past yield data not obtained by government or researches [32–34], although the information gathered through LEK has not been readily accepted [35]. Today, there is a growing recognition that fishers’ LEK could fill up gaps in biological, ecological and management knowledge [30, 34, 36, 37], as long as there is some caution in interpreting its quality and accuracy to science [32]. Fishers may provide accurate information on fish diet, for instance [38], which does not exempt them from misinterpreting such information. For example, for observing that a given fish has once in a while been caught with lobster in its stomach, fisher may seem such fish as a competitor capable of affecting their lobster fishing [39]. Therefore, it is important to identify the type of biological information that fishers can reliabily provide, which will depend on social, economic and local ecological factors. Second, it is also relevant to interpret such information through a scientific filter.

Finally, the large extension of marine ecosystems makes it difficult, financially and technically, to gather detailed scientific information about them along the time [17, 40]. On the other hand, fishers’ LEK can cover large coastal and offshore areas and can also track changes over large temporal scales, potentially minimizing costs and improving the management success [17, 31, 41]. Considering such potentiality, some studies have suggested the need of adopting fisheries co-management systems that integrate fishers and their knowledge to the scientific knowledge and to the political management process usually done in partnership with goverments [42, 43]. The success of such initiatives depend on multiple factors, one of them being the inclusion of open-minded scientists capable of valuing fishers’ LEK to establish management goals and enforcement mechanisms [17], in a learning by doing process [44].

In this study, we assessed fishers’ LEK and a more conventional fishery approach, here described as scientific knowledge (SK), to know if LEK could yield reliable and accurate information regarding catch patterns and regarding the environmental influence on fishing decisions. More specifically, we hypothetised that fishers choose their fishing spots not only based on the spot productivity, but also on the perception of environmental factors that limit fishing. We specifically assessed the seasonal and bathymetric patterns of species caught by small-scale fishers through two different approaches: (1) on-board scientific monitoring of small-scale fisheries, and (2) structured interviews with fishers about their local ecological knowledge of fishing regarding the occurrence and catch biomass of target species. Such methods were assessed for their differences and complementarities, as an evaluation of the possibility of using LEK to provide spatial-temporal data on catch, whenever more traditional scientific approaches are not an option.

## Methods

### Study area

**Fig. 1:**
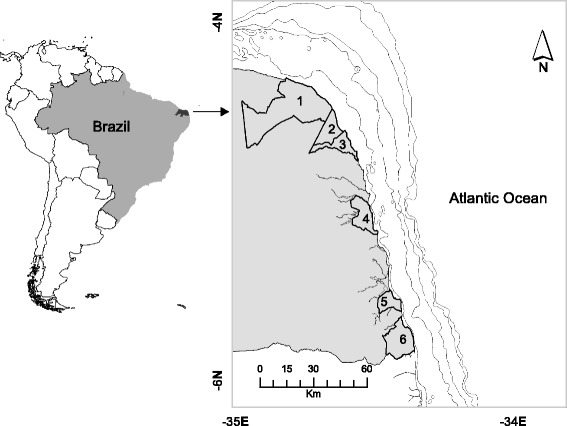
Map of the study area. Municipalities are highlighted by the limits and numbered in ascending order from N to S. Touros – 1, Rio do Fogo – 2, Maxaranguape – 3, Natal – 4, Tibau do Sul – 5 and Baía Formosa – 6

Six municipalities of the eastern coast of Rio Grande do Norte, a coastal state on the Brazilian northeast, were sampled (Fig. 1). Such places were chosen because they cover an important region where bottom-set gillnets are used by commercial small-scale fisheries. Such places responded for an average of 78.4% of the catch of the entire state, between 2011 and 2014, with a minimum of 76.8% in 2014 and a maximum of 81.9% in 2011 (unpublished data provided by the “Sea around Us” reconstruction effort).

The study region is marked by a rainy season from February to July, and a dry season from September to January. The southeast winds are more frequent and stronger between April and July, and are usually accompanied by rain [45]. During rainfall periods, the ocean currents and southeast winds produce 1.5 m average height waves that influence the entire continental shelf [46, 47].

Throughout the year, the small-scale fishing fleet performs round trips with boats powered mostly by sail and by small one-cylinder motors. Most do not have supporting equipment to fishing and navigation and the fishing trips last between one and four days [48]. In fact, most of the trips are completed within the same day. Even when longer, these trips are not usually done to places further away from the coast. The average distance for fishing trips in the region is 10 km off the coast.

The bottom-set gillnets used in the study region generally have two different settings. The first one usually has smaller mesh size, which is referred by fishers as a “fine mesh”, varying from 80 to 100 mm (opposite knot) with a nylon thickness varying from 0.40 to 0.60 mm. The net total length varies between 500 and 2,500 m and has an average height of 1.6 m. This first setting is used throughout the year and is cast mainly close to reefs and over the biodetrical substrate (rhodolith of calcareous algae and carbonate debris from marine organisms), remaining in the water for around 3.5 h. This setting is used anywhere on the continental shelf. The second kind of bottom-set gillnet has a larger mesh, called “thick net”, and is used mostly during the spring and summer. Its mesh size ranges between 120 and 320 mm and the length between 558 and 2,100 m. Such nets are set mainly on muddy shallow substrates (5–20 m), remaining submerged for about 5 h.

### Data collection

The data collection was divided in two phases. The first phase consisted in obtaining information on Scientific Knowledge (SK) through on-board observations of the fleet that used bottom-set gillnets between June 2012 and June 2014. The second phase consisted in registering Fishers’ Knowledge (LEK) by using semi-structured interviews applied only to fishing masters and active expert fishers that were using bottom-set gillnets during the study period. The second phase was refined after collecting SK data and was done between August 2014 and January 2015. The data collection could not be simultaneous because the on-board observations were used to identify difficulties and solutions implemented by fishers when at sea, during certain periods of the year or in specific geographical areas. Such observations drove the development of the second phase (LEK).

For the SK phase, only data coming from small motorized boats (8 a 9 m) that used “fine” mesh size (80–100 mm opposite knot) were considered. The fleet with such characteristics responds for most of the fishing effort in the area. A total of 72 net settings were registered, under the criteria defined above.

The number of respondents for the LEK phase was calculated beforehand, by visiting each municipality to estimate the number of boats and of active fishers. In each place several fishers were asked the number of vessels using bottom-set gillnet and the average crew size per boat. The fishers’ responses were averaged out to estimate the total number of fishers working in bottom-set gillnet boats. That resulted in an estimate of 28 boats and 56 fishers. Of those, 32 skilled fishers (according to their peers) were interviewed, which were all fishing in boats between 8 and 9 m long and using fine meshes. Most (79% of 32) interviewees were fishing masters; of these, 13 had also been followed previously during the on-board data collection phase. Such preference for fishing masters was due to their accumulated knowledge. These are usually the most experienced fishers in the crew and the ones making decisions regarding where to go and how long to stay on each spot. Overall, the respondents were experienced fishers (average = 30 years as a fisher, 22 years using bottom-set gillnet), even though they were relativey young (most were between 35 and 58 years old). These fishers tended to have a low level of education (12% were illiterate and 88% did not complete primary school) and low income (62% lived on less than the minimum wage).

We only interviewed fishers after carefully explaining the goal of our research personally and individually and after they gave their oral consent to take part in the study. We explained that they had the right to refuse participation, but no fisher we approached refused to join the study.

### Scientific knowledge (SK) – On-board data

Every time a gillnet was set, three sets of variables were recorded: (1) geographical, which included geographic location and the average depth of the setting site; (2) biological, including species identification and total catch (kg) per species; and (3) fishing related variables, including net length and height, mesh size and time the net spent in the water (soaking time).

The fishing related variables were used to calculate the fishing effort (F):

1 $$ \mathbf{F} = \mathbf{A}\ *\ \mathbf{T} $$

Where “A” represents the gillnet area in square meters (height * total length); and “T” is the soaking time (in hours). “F” is presented as m^2^ *h.

The fishing effort was then used to calculate the CPUE (Capture per Unit Effort), which is an indirect measure of local fish abundance. The CPUE was defined as the ratio between catch and effort and calculated for both methods (LEK and SK), with the same fishing effort parameters:

2 $$ \mathrm{C}\mathrm{PUE} = \mathrm{C}/\mathrm{F} $$

Where C is the catch in weight per sample (fish caught per cast of a bottom-set gillnet) and F is the fishing effort (eq. 1). The CPUE is then presented as g/m^2^*h. The CPUE was calculated for the different depths and different species considered in this study.

### Interviews with fishers - LEK

During the on-board observation, it was noted that: 1) a few species, namely blue runner [*Caranx crysos* (Mitchill, 1815)], serra Spanish mackerel (*Scomberomorus brasiliensis* Collette, Russo & Zavala-Camin, 1978), and lane snapper [*Lutjanus synagris* (Linnaeus, 1758)], comprised most of the catch; and that 2) fishing seemed limited by local environmental conditions, such as the current strength and the presence of drifting algae. Such preliminary observations of possible environmental factors limiting the fishing operation at different depths and periods of the year drove the design of the LEK interview, which also focused on the three main target species.

The interviews had quantitative and qualitative questions regarding fishers’ perception about the fishing operation, gillnet measures, how they define what is deep and shallow fishing areas (in meters), and soaking time (in hours). Such information was used to calculate fishing effort and CPUE, using the same definition presented before (Eqs. 1 and 2). The interviews also approached the depth of fishing, average weight of the catch and the havesting period for each of the three species (Additional file 1). Similarly to the SK approach, such information (effort and harvest) was used to calculate the CPUE per depth and species.

Instead of directing fishers to specific environmental factors assumed to be relevant to affect their fishing, fishers were simply asked what environmental factors they considered adverse for fishing. For each factor that the fishers mentioned, they were inquired about the period of the year when it most commonly happens. As expected, based on on-board observations, strong currents and large amounts of drifting algae were the main factors mentioned by the fishers (62% mentioned at least one of these factors). Fishers said that these two factors hamper fishing operations and can even result in the loss of gillnets. Therefore, only data for these two environmental factors are presented. The fishers were also asked when each of the three species was most abundant along the year.

Fishers were asked about periods in two moments of the interview: the periods of adverse environmental factors to fishing and the peak periods for catching the three target species. If, instead of answering a specific month(s) for either of these questions, the interviewee answered with a season (dry or wet season), he was asked to identify the months that were most typically considered dry and wet, in his own perception.

### Data analysis

**Fig. 2:**
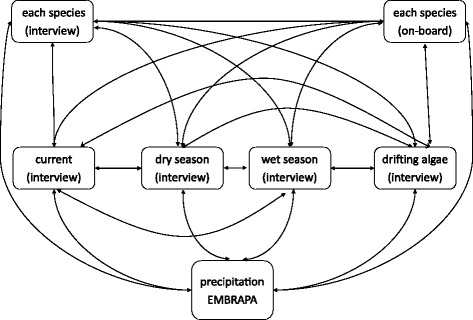
Pearson correlations (r) run in the study

We used the number of times the fishers cited a given month to calculate the seasonal frequency of the environmental factors (currents and drifting algae) affecting fisheries and of when each of the three target species were considered more abundant. For instance, if a fisher said that wet months were those between April and August, these months received “1”, and the remaining months “0”. This was done for every fisher and for the five questions regarding: wet season, dry season, months of strong currents, months of drifting algae, and months of higher catches for the three main species. We used the Pearson correlation method to check the relationship between the absolute frequency of answers each month got with the incidence of environmental factors and occurrences (also measured as number of times cited) of the species (Fig. 2). The precipitation pattern was provided by EMBRAPA (Brazilian Agricultural Research Corporation) for the period 2012 June to 2014 June.

The presence of a species at different depths (6 to 50 m) was correlated between LEK and SK by the Spearman correlation test, with depths paired and sorted in ascending order. We also evaluated if the occurrence of a species at a given depth was correlated amongst methods (LEK and SK).

Another Spearman correlation was done to verify if the depths of larger and smaller CPUE are correlated amongst methods (LEK and SK). This was done after ordering the CPUE from the highest to the lowest value.

Due to the differences in biomass scales obtained between methods (fishers tend to cite much higher catches in the LEK method than what was observed in the SK), the CPUE was transformed by log_10_(CPUE + 1) to make them comparable, as the idea was to compare catch patterns and not absolute values by depth between methods.

## Results

### Seasonal patterns

The fishing catches analyzed on site resulted in 3,732 fish individuals of 93 species, totalling 2193.3 kg. As specified earlier, three species were the most common and most abundant in the catches: blue runner (*Caranx crysos =* 16%), serra Spanish mackerel (*Scomberomorus brasiliensis* = 11%) and lane snapper (*Lutjanus synagris* = 7%), representing approximately 37% of the individuals and 34% of the catch weight.

**Fig. 3:**
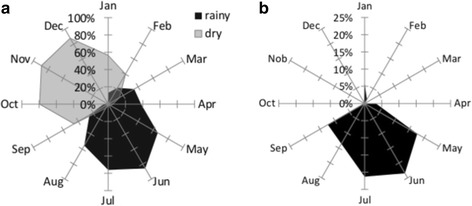
Relative frequency of rainfall in the year, as perceived by fishers (**a**) and recorded monthly by the local meteorological institution between 2012 and 2014 (EMPARN) (**b**)

The fishers were accurate at recognizing the rainy season (Fig. 3a), which was observed by the high correlation between the months they mentioned and the most intense period of rainfall (between May and July) observed between 2012 and 2014 (r = 0.90; *p* = 4,82E-05) (Fig. 3a and b; Table 1). Most of them (62%) cited that the rainy season is also when currents are the strongest and when drifting algae are more abundant (Table 1).

**Table 1:** Significant Pearson correlations. All fish data refer to absolute frequency

Method	Correlation	Peason (r)	Value *p*
LEK	rainy x mackerel	0.864	0.000292
	rainy x current	0.935	8.43E-06
	rainy x algae	0.951	2.03E-06
	rainy x precipitation	0.906	4.82E-05
	mackerel x current	0.96	7.39E-07
	mackerel x algae	0.946	3.19E-06
	mackerel x precipitation	0.925	1.67E-05
	snapper x dry	0.81	0.0015
	current x algae	0.948	2.82E-06
	current x precipitation	0.901	6.19E-05
	algae x precipitation	0.948	2.73E-06
SK	runner x mackerel	0.747	0.012973
	mackerel x precipitation	0.698	0.024701

The season for serra Spanish mackerel, according to the fishers, was correlated both with their identification of the rainy season and with the actual registered rainy season. The presence of this species was also correlated with fishers’ perception of drifting algae and strong currents periods.

**Fig. 4:**
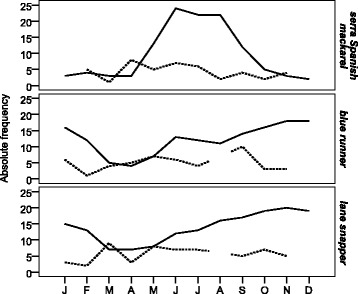
Monthly occurrence of the three main species. The solid line represents the catches reported by fishers (LEK), and the dotted line represents catches actually registered on board (SK)

Fishers established clear occurrence patterns for the three species, which was confirmed for two of them (Table 1). The serra Spanish mackerel was expected by them to be more abundant in the rainy season, whereas the blue runner was expected to present two peaks of abundance, one in the dry season up to December and another in June, the peak of the rainy season. Lane snapper was expected to be majorly abundant in the dry season, although some fishers mentioned its occurrence in the rainy season as well (Fig. 4).

On the other hand, when variables classified as SK were correlated between themselves (see Fig. 2), only the correlation between precipitation and frequency of serra Spanish mackerel in the fishing confirmed that indeed this species was more common in the rainy season, as previously suggested by fishers. The SK correlations suggested an additional correlation between serra Spanish mackerel and blue runner.

### Spatial patterns

**Fig. 5:**
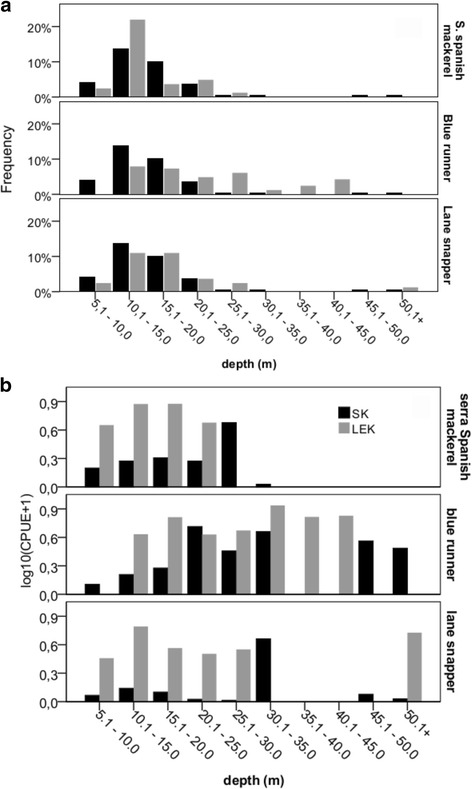
Bathymetric patterns of species distribution in occurrence (**a**) and CPUE (**b**), based on information obtained from fishers (LEK) and from on-board observations (SK)

The occurrence of these species at different fishing depths, as registered on board, was significantly correlated to fishers’ information (citations of the depth range where each species occurs) (serra Spanish mackerel r = 0.98, *p* = 9.7E-044; blue runner, r = 0.99, *p* = 2.1E-059; lane snapper, r = 0.99, *p* = 3.8E-055). Therefore, fishers were accurate at reporting the depth that each of these species would be more commonly found. Both fishers’ information and actual fishing observation suggest a concentration of boats in shallower waters, among the isobaths of 10 and 20 m (Fig. 5a). Also, for the fishers, currents get stronger and algae become more abundant with increasing depths (r = 0.93, *p* = 9.253E-006; r = 0.88, *p* = 1.498E-004, respectively). Such fact limits their fishing to shallower waters (95% of them did not use deeper areas), even though the fishers claimed that deeper waters are best for fishing (*X* ^2^ = 15.36; *p* = 8.88E-05).

On the other hand, the CPUE estimated from fishers’ information was significantly different from the one calculated from on-board observations (*T*-test serra Spanish mackerel *p* = 0.002; blue runner *p* = 0.001; lane snapper *p* = 1.3E-005). Fishers’ estimates resulted in higher means and standard deviations than the actual CPUE, even after transformation of the data (Table 2).

**Table 2:** Variation of the CPUE (Kg/Km^2^*h) for the three main species, according to fishers’ information and according to data registered on board

	On-board records			Fishers’ information		
	serra Spanish mackerel	blue runner	lane snapper	serra Spanish mackerel	blue runner	lane snapper
Number of samples	39	45	52	28	27	26
Minimun	0.07	0,06	0.03	0.33	0.29	0.19
Maximum	5.77	18.87	3.59	36.27	41.67	17.72
Mean	1.17	1.75	0.37	9.66	9.64	4.68
Stand. deviation	1.32	3.62	0.51	9.21	10.51	4.13

When the values of CPUE by depth were ranked from the highest to the lowest, higher values of CPUE were reported for deeper areas in both methods, with significant correlations between the fishers’ information and the observations on-board for the most representative species.

The correlation of CPUE per depth between methods was higher for more valuable commercial species. Serra Spanish mackerel is the most sought species because of its quality and abundance (r = 0.473; *p* = 0.00004), whereas lane snapper is the priciest and is present more frequently in the fishing, but is the least abundant in catches (r = 0.297; *p* = 0.012). On the other hand blue runner was weakly correlated between methods. This species had the highest abundance observed in catches, but the lowest commercial value.

On-board observations agreed with fishers’ claim that stated that fishing frequency decreased with increasing depth after 20 m. On the other hand, the CPUE of the three analyzed species were larger in deeper areas (Fig. 5a and b).

### Overlapping spatial information

A complementary pattern was observed when both methods were overlapped. Blue runner had the widest bathymetric distribution, occurring from 10 to 50 m deep. For fishers, the CPUE of this species increased up to intermediate depths, between 30 and 40 m, whereas on-board data suggested a decrease in the CPUE after 50 m. (Fig. 5b). The observed bathymetric occurrence of lane snappers also coincided with what fishers suggested, especially regarding the interval between 10 and 20 m deep. The CPUE showed no clearly defined bathymetric patterns. However, both fishers and actual observation suggested that larger catches of lane snapper were observed around 30 m deep. According to fishers this species often occurs in catches with bottom-set gillnets, although the catches are not usually large. Neither of the methods provided a reliable pattern for the isobaths between 30 and 40 m for both the blue runner and the lane snapper, presumably due to the low frequency of fishing in such deeper areas (Fig. 5b). Serra Spanish mackerel, which showed congruent information for both methods regarding occurrence and CPUE, was mentioned by fishers and also confirmed by on-board observation to have its highest catches in waters 20 m deep.

## Discussion

This study shows that the use of local ecological knowledge (LEK) approach can provide reliable information on fish seasonal and spatial patterns equivalent to conventional scientific methods in fisheries science, although with somewhat wider variation. Besides, such approach is less time-consuming and less expensive than the traditional ones used in fishery sciences [34], and not limited to the direct observation of fishing operations or fish landing sampling.

### Seasonal patterns

The temporal record of fishing that fishers can offer provides a wealth of accurate details to understand the dynamics of fisheries and of environmental marine resources. This method, even if applied to few interviewees, can identify seasonal patterns for target species, which in this case was clear mostly for serra Spanish mackerel and lane snapper. On the other hand, on-board data, when done along a short period of time, may not be enough to establish seasonal patterns, probably because, in this case study, bottom-set gillnet is a multi-specific gear and also because there is wide variation in seasonality from year to year, affecting species patterns in the short term [49].

Fishers’ knowledge tends to be spatially localized and seasonal. It is primarily acquired during observations that take place on fishing grounds during a given fishing season [50]. The accumulated experiences of fishers probably reflect the seasonal patterns accurately. The experience shared by many fishers on a daily base helps them build consensus, which can be extracted even from a small number of informants [51]. These shared experiences are based on a wide range of trials and errors adjusted individually and collectively to optimize catches with lower fishing effort.

The seasonal patterns of species occurrence described in the literature and fishers’ knowledge gathered here are in agreement. The higher occurrence of blue runner and lane snapper in the dry season is related to their reproductive period, which goes from early spring to late summer, as noted in the Mediterranean [52] and in Venezuela [53]. For the serra Spanish mackerel, their migration has been related to feeding during the dry season (March to August) in northern Brazil [54] and to reproduction during the rainy season (May to August) in the northeastern Brazilian coast [48].

Fish abundance is always changing: populations are patchily distributed and such distribution, depending on the scale, can vary daily, seasonally, inter-annually, and from decade to decade in relation to naturally varying conditions, as well as in response to human influences. Isolated observations may therefore have little value in evaluating change [55], in which case the long-term observations provided by fishers may be useful, as long as potential biases are considered. For example, fishers’ knowledge has been shown to be more accurate when reporting extreme positive events related to abundance, such as their best individual catch ever for each species [32].

### Spatial patterns

Here, it is also shown that fishers can provide reliable data regarding patterns of occurrence and catches at different depths, as this information was confirmed by on-board observations. Confirming one of the hypotheses, fishers did perceive deeper areas as more productive, but fishing in such areas was limited by strong currents and the presence of drifting algae, forcing fishers to use shallower and less productive waters. Currents have known influences on the oceanographic dynamics of the continental shelf [46, 47]. One such influence is likely to be changes in the abundance of drifting algae [56, 57], although this is not known in the literature for the studied coast. The effects of drifting algae on fisheries are not known in the global literature either. Only through LEK it was possible to understand the choice fishers make when chosing their grounds, reinforcing the idea that some areas are protected against overexploitation by natural and technological circumstances, such as boat size, inaccessibility and by adverse enviromental factors [58].

According to the fishers, strong currents force the gillnets onto the ground, which results in considerable loss of their catches, besides making it difficult to pull the net back. In the event of very strong currents, nets can be ripped or even lost. On the other hand, drifting algae do not affect catches as much as it affects the difficulty of pulling the net back, due to their extra weight. In both cases the problems are intensified in deeper waters. However, it was not possible to quantify such influence during the on-board observations.

The high occurrence of strong currents and drifting algae in deeper areas was probably responsible for the fewer observed net settings in these areas, despite the fact that both the observed data and fishers’ LEK suggested that these were more productive sites. This is especially clear for blue runner, which was not caught between the 30 and 50 isobaths, but was mentioned by fishers to be productive in such depths. However, for the three species analyzed there is either no (serra Spanish mackerel) or very little information (blue runner and lane snapper) to confirm if such fishes are really not abundant in depths above 30 m, as fishers rarely fished in such areas. As expected, fishers rarely gave information regarding productivity in such depths.

Fishing at intermediary depths is usually unrelated to the fishing gear used, but associated to the ecological characteristics of the species being targeted [59]. For instance, in the Baleares Islands in the Mediterranean, fishers trawl at the slope of the continental shelf due to the biological features of the target species, which is more abundant in such depths [59]. Another study also suggested higher CPUE at 25–50 m deep for the yellow tail snapper *Ocyurus chrysurus* (Bloch, 1791) caught by small-scale fisheries using hook and line on the Brazilian northeast [60]. Such results led the authors to suggest that intermediary depths should be managed to protect this snapper.

### Overlapping approaches

Fishers are a recognized source of marine knowledge, and they need to be involved in the collection and evaluation of their experience in fishery management [61]. Scientists should consider fishers’ knowledge, especially when scientific knowledge about ecosystem functions is insufficient to provide unambiguous answers to management problems [62]. The complementarity of SK and LEK approaches generate more robust information and that could lead us a step further in the decision-making process. Some researches believe that understanding key social-ecological linkages could support a transition toward sustainability in small-scale fisheries. Such claim is based on the importance of partnerships that transcend disciplines and conventional approaches involving multiple stakeholders to work collaboratively toward sustainable strategies [63, 64].

The recovery of the fishers’ memories reported here was generally consistent with what was observed on board (high biomass at intermediate depths), besides providing additional information not available scientifically (seasonality of species and limited fishing areas caused by enviromental factors). Even though the onboard data comprised short time frame and interviews are somewhat subjectives for relying on people’s memories, they presented convergent and reliable information regarding the concentration of fishing effort mostly in shallower depths. This approach has the advantage of incorporating unreported catches that would be otherwise lost in conventional methods [34].

Fishers were more accurate at describing the bathymetric pattern of CPUE for species of high commercial value, namely serra Spanish mackerel and lane snapper. For the blue runner, the methods (SK and LEK) did not agree, even though this was an abundant species in the catches. Perhaps, its cheaper price makes less of a memorable impression than its large catches. There is some evidence that fishers’ perception is more accurate for commercial species or at least those with remarkable large and recent catches [32]. Researchers agree that a systematic approach during the interviews is crucial. Fishers tend to feel more at easy when they are asked specific questions, such as about an exceptional event (e.g., their best catch ever) or about a typical catch at different times of their lives or at different seasons, rather than vague questions (e.g., how much the catch has changed) [34, 65].

However, even if such care is taken into the formulation of a question, fishers seem to overestimate typical catches, whereas they tend to recall their best catch better. This is attributed to a common cognitive phenomenon, known as “flashbulb” memory, which are those past events with personal importance and/or with striking consequences [66]. On the other hand, the typical catch, although more recurrent, may be hard to be accessed. However the approach of patterns in the typical catches data used in this study showed consistent information about spatial and temporal distribution of main species caught with bottom-set gill net.

It is also important to acknowledge the relevance of considering the expertise of those being interviewed. Fishers’ experience depends on several factors such as their years of active fishing, gear and exploited area [36, 61]. For this reason, in the present study knowledgable fishers and fishing masters were the only ones interviewed. Specifically, fishing masters, who comprised most of the sample, tend to own the nets, and are responsible for choosing the nets to be used, which determine where those will be set and which fishing resources will be targeted. Therefore, their expertise encompasses a very particular and detailed knowledge of the local environmental conditions and of the ecological relations happening on a given site [36]. Here specifically it has been shown that fishers that have been fishing for about 30 years can identify seasonal and spatial patterns of occurrence of their main target species. However, it is not possible to confirm that less experienced fishers would not provide the same type of information, as this information was not compared across ages.

Finally, the integration of different types of knowledge has direct implications for an EAF. For instance, if all the information used in management were based on SK, it would be known that most effort is concentrated in shallower waters, and therefore such areas would require specific protection. Whereas ecological appropriate, such measures would be socially harmful [67], since the fact that fishers limit their effort to shallow waters is not necessarily because these are productive grounds, but because they face technological and ecological limitations. Such information could only be known through LEK, reinforcing the need of having integrative and participatory management systems [30, 43, 44, 68, 69].

## Conclusions

This study showed that the integration of conventional fishing approaches with experiences accumulated by fishers reveal a great influence of seasonal and spatial dynamics of marine environmental factors causing higher fishing pressure in shallow and usually poorer waters. Besides, it revealed the importance of having direct on-board observation not only to produce more realistic and detailed data, but also as a way to confirm the factors that hamper fishing operations. Once established the accuracy of LEK in relation to SK, LEK may be used to gather a set of reliable information for fisheries management through well-structured interviews capable of quickly revealing ecological patterns of target species. This is not to say that one type of knowledge is superior to another, but that their integration might be the best path for fisheries sustainability. Translating LEK into an accessible language to scientists is likely also an important step to achieve its integration into management and to provide a more holistic and more realistic understanding of fishing. We therefore advocate for a continuous policy of fish landing sampling that contemplates effort data on bathymetric and general oceanographic conditions, but that also includes LEK to understand how such conditions interfere with fisheries.

Specifically, the fishing patterns observed in areas less exploited due to environmental limitations are important in fishing zone selection for the management of fishing bottom set gillnet and to prevent the emergence of ghost nets caused by the loss of nets on the seabed that continue killing marine organisms indefinitely. These patterns need to be further investigated by joining fishers and landing observations over large spatial and temporal scales. Besides, additional research should use LEK to identify other environmental limitants on fishing effort and production that could be used as stepstones to management.

The results showed here confirm that fishers do detain an important body of knowledge that could support faster and more affordable management initiatives. Moreover, fishers could certainly contribute with additional information where there is no official statistics. As science advances, it becomes clearer that fishers can enhance our understanding of marine ecosystem dynamics and of fisheries in general, which is not easily or cheaply achieved solely by conventional approaches.

## Additional file

Additional file 1: Interview form (DOCX 36 kb).
