# Supplementary material for: The use of Local Ecological Knowledge as a complementary approach to understand the temporal and spatial patterns of fishery resources distribution
Source: J Ethnobiol Ethnomed. 2017 Jun 1;13:30. doi: 10.1186/s13002-017-0156-9 (PMC5455079; doi:10.1186/s13002-017-0156-9)
Supplement: Additional file 1: — Interview form (DOCX 36 kb). [file 13002_2017_156_MOESM1_ESM.docx]

**Supplementary Material - interview form**

1- how old are you?

2- When did you start fishing?

3- When did you start fishing using gillnets?

4- What is the length and height of your current net? Size of the stretched mesh? How many hours do you usually leave the net soaking in the water?

5- How deep is the deepest fishing area you usually fish?

6- How deep is the shallowest fishing area that you usually fish?

7- What are the rainy and dry months? rainy months - J F M A M J J A S O N D; dry months - J F M A M J J A S O N D

8- Which months have the strongest currents? Months - J F M A M J J A S O N D

9- Which months have the largest amounts of drifting algae? Months - J F M A M J J A S O N D

10- What months present the highest catches of blue runner? Months - J F M A M J J A S O N D

11- What is the depth range that blue runner occusr?

12- On average, what is the regular catch (kg) of blue runner in shallow areas during the rainy season? _____ And during the dry season? ______

13- - On average, what is the regular catch (kg) of blue runner in deep areas during the rainy season? _____ And during the dry season? ______14- What months present the highest catches of serra spanish mackarel? Months - J F M A M J J A S O N D

16- What is the depth range that serra spanish mackarel occurs?

17- On average, what is the regular catch (kg) of serra spanish mackerel in shallow areas during the rainy season? _____ And during the dry season?

18- On average, what is the regular catch (kg) of serra spanish mackerel in deep areas during the rainy season? _____ And during the dry season?______

19- What months present the highest catches of lane snapper? Months - J F M A M J J A S O N D

20- What is the depth range that lane snapper occurs?

21- On average, what is the regular catch (kg) of lane snapper in shallow areas during the rainy season? _____ And during the dry season?

22- On average, what is the regular catch (kg) of lane snapper in deep areas during the rainy season? _____ And during the dry season?

**Supplementary Material - On-board data**

We registered 102 species, ranked in descending order below. The three main ones (total catch) were selected for the study.

|  |  |  | **Weight (g)** | | |
| --- | --- | --- | --- | --- | --- |
| **Species** | **Common name** | **n** | **Total** | **Mean** | **Stan. Dev.** |
| *Caranx crysos* | Blue runner | 775 | 381.995 | 492,9 | 139.3 |
| *Scomberomorus brasiliensis* | Serra Spanish mackerel | 360 | 253.816 | 705 | 277.1 |
| *Euthynnus alletteratus* | Little tunny | 195 | 190.200 | 975,4 | 362.7 |
| *Haemulon parra* | Sailor's grunt | 489 | 165.080 | 337,6 | 104.4 |
| *Lutjanus synagris* | Lane snapper | 354 | 124.280 | 351,1 | 141.3 |
| *Sphyrna lewini* | Scalloped hammerhead | 2 | 100.171 | 50085,5 | 7858.1 |
| *Centropomus undecimalis* | Common snook | 38 | 99.907 | 2629,1 | 594 |
| *Rhizoprionodon porosus* | Caribbean sharpnose shark | 74 | 93.348 | 1261,5 | 1079.5 |
| *Bagre marinus* | Gafftopsail sea catfish | 153 | 85.569 | 559,3 | 258.2 |
| *Carcharhinus acronotus* | Blacknose shark | 9 | 82.370 | 9152,2 | 4162.5 |
| *Acanthurus chirurgus* | Doctorfish | 309 | 67.406 | 218,1 | 65.2 |
| *Haemulon plumierii* | White grunt | 212 | 54.772 | 255,1 | 74.5 |
| *Caranx bartholomaei* | Yellow jack | 91 | 48.747 | 535,7 | 222.7 |
| *Sciades proops* | Crucifix sea catfish | 90 | 47.150 | 523,9 | 179.8 |
| *Sparisoma axillare* | Gray parrotfish | 84 | 39.303 | 467,9 | 140.7 |
| *Aspistor luniscutis* |  | 64 | 37.746 | 589,8 | 262.4 |
| *Carcharhinus limbatus* | Blacktip shark | 1 | 36.031 | 36031 |  |
| *Echeneis neucratoides* | Whitefin sharksucker | 39 | 31.949 | 819,2 | 364.9 |
| *Elops saurus* | Ladyfish | 29 | 31.765 | 1095,3 | 293 |
| *Rachycentron canadum* | Cobia | 17 | 31.606 | 1859,2 | 3993 |
| *Auxis thazard* | Frigate tuna | 43 | 24.757 | 575,7 | 240.8 |
| *Echeneis naucrates* | Live sharksucker | 32 | 24.229 | 757,2 | 374.1 |
| *Bagre bagre* | Coco sea catfish | 49 | 21.185 | 432,3 | 111 |
| *Lutjanus chrysurus* | Yellowtail snapper | 47 | 21.001 | 446,8 | 104.4 |
| *Caranx lattus* | Horse-eye jack | 18 | 19.849 | 1102,7 | 952.1 |
| *Lutjanus analis* | Mutton snapper | 20 | 19.554 | 977,7 | 728.3 |
| *Sparisoma frondosum* | Agassiz's parrotfish | 34 | 16.085 | 473,1 | 201.6 |
| *Anisotremus virginicus* | Porkfish | 58 | 13.338 | 230 | 131.1 |
| *Scomberomorus cavalla* | King mackerel | 14 | 13.119 | 937,1 | 667.7 |
| *Holocentrus ascensionis* | Squirrelfish | 78 | 12.546 | 160,8 | 37.7 |
| *Priacanthus arenatus* | Atlantic bigeye | 58 | 12.536 | 216,1 | 112.3 |
| *Opisthonema oglinum* | Atlantic thread herring | 81 | 12.230 | 151 | 70.9 |
| *Micropogonias furnieri* | Whitemouth croaker | 10 | 10.213 | 1021,3 | 182.3 |
| *Albula vulpes* | Bonefish | 20 | 10.167 | 508,4 | 290 |
| *Scomberomorus regalis* | Cero | 15 | 9.938 | 662,5 | 309.5 |
| *Cynoscion leiarchus* | Smooth weakfish | 7 | 8.080 | 1154,3 | 1023.8 |
| *Mycteroperca bonaci* | Black grouper | 1 | 7.820 |  |  |
| *Elagatis bipinnulatus* | Rainbow runner | 5 | 6.380 | 1276 | 137.4 |
| *Seriola rivoliana* | Longfin yellowtail | 9 | 6.123 | 680,3 | 179.6 |
| *Lycengraulis grossidens* | Atlantic sabretooth anchovy | 6 | 6.101 | 1016,8 | 460.4 |
| *Calamus penna* | Sheepshead porgy | 27 | 5.823 | 215,7 | 71.5 |
| *Caranx hippos* | Crevalle jack | 1 | 4.795 |  |  |
| *Calamus pennatula* | Pluma porgy | 15 | 4.655 | 310,3 | 420.3 |
| *Chaetodipterus faber* | Atlantic spadefish | 12 | 4.417 | 368,1 | 154.3 |
| *Haemulon aurolineatum* | Tomtate grunt | 28 | 3.816 | 136,3 | 84.6 |
| *Sparisoma amplum* | Reef parrotfish | 5 | 3.595 | 719 | 179.4 |
| *Cynoscion virescens* | Green weakfish | 1 | 3.206 |  |  |
| *Conodon nobilis* | Barred grunt | 15 | 3.201 | 213,4 | 44.3 |
| *Lactophrys trigonus* | Buffalo trunkfish | 3 | 3.158 | 1052,7 | 443.8 |
| *Chloroscombrus chrysurus* | Atlantic bumper | 28 | 2.934 | 104,8 | 30.1 |
| *Syacium papillosum* | Dusky flounder | 22 | 2.636 | 119,8 | 33.9 |
| *Heteropriacanthus cruentatus* | Glasseye | 10 | 2.428 | 242,8 | 78.9 |
| *Lutjanus alexandrei* | Brazilian snapper | 6 | 2.425 | 404,2 | 29.9 |
| *Selene brownii* | Caribbean moonfish | 9 | 2.392 | 265,8 | 251.8 |
| *Selar crumenophthalmus* | Bigeye scad | 18 | 2.356 | 130,9 | 26.3 |
| *Dactylopterus volitans* | Flying gurnard | 10 | 2.353 | 235,3 | 103.7 |
| *Fistularia tabacaria* | Cornetfish | 1 | 2.020 |  |  |
| *Rhinobatus percellens* | Chola guitarfish | 4 | 2.013 | 503,3 | 134.3 |
| *Lutjanus jocu* | Dog snapper | 3 | 1.920 | 640 | 337.9 |
| *Decapterus tabl* | Redtail scad | 10 | 1.900 | 190 | 55.2 |
| *Uraspis secunda* | Cottonmouth jack | 2 | 1.855 | 927,5 | 102.5 |
| *Pomacanthus paru* | French angelfish | 2 | 1.825 | 912,5 | 866.2 |
| *Gymnothorax moringa* | Spotted moray | 2 | 1.812 | 906 | 33.9 |
| *Trachinocephalus myops* | Snakefish | 10 | 1.806 | 180,6 | 82.8 |
| *Scorpaena plumieri* | Spotted scorpionfish | 5 | 1.805 | 361 | 206.6 |
| *Dasyatis marianae* | Brazilian large-eyed stingray | 2 | 1.594 | 797 | 414.4 |
| *Mugil curema* | White mullet | 3 | 1.573 | 524,3 | 111.5 |
| *Centropomus parallelus* | Fat snook | 1 | 1.560 |  |  |
| *Acanthurus bahianus* | Ocean surgeon | 7 | 1.360 | 194,3 | 30.9 |
| *Acanthostracion quadricornis* | Scrawled cowfish | 8 | 1.308 | 163,5 | 79.7 |
| *Trichyurus lepturus* | Largehead hairtail | 1 | 1.282 |  |  |
| *Lagocephalus laevigatus* | Smooth puffer | 1 | 1.257 |  |  |
| *Oligoplites palometa* | Maracaibo leatherjacket | 1 | 1.250 |  |  |
| *Selene vomer* | Lookdown | 3 | 1.189 | 396,3 | 498.6 |
| *Synodus intermedius* | Sand diver | 5 | 1.090 | 218 | 66.5 |
| *Archosargus rhomboidalis* | Western Atlantic seabream | 4 | 1.020 | 255 | 107.9 |
| *Oligoplites saliens* | Castin leatherjacket | 1 | 905 |  |  |
| *Sphyraena guachancho* | Guachanche barracuda | 1 | 821 |  |  |
| *Fistularia petimba* | Red cornetfish | 1 | 790 |  |  |
| *Syacium micrurum* | Channel flounder | 7 | 788 | 112,6 | 30.9 |
| *Notarius grandicassis* | Thomas sea catfish | 1 | 760 |  |  |
| *Ulaema lefroyi* | Mottled mojarra | 12 | 698 | 58,2 | 19.1 |
| *Alphestes afer* | Mutton hamlet | 2 | 659 | 329,5 | 152 |
| *Gymnothorax vicinus* | Purplemouth moray | 1 | 592 |  |  |
| *Haemulon steindachneri* | Chere-chere grunt | 6 | 530 | 88,3 | 42.4 |
| *Mulloidichthys martinicus* | Yellow goatfish | 2 | 510 | 255 | 7.1 |
| *Orthopristis ruber* | Corocoro grunt | 3 | 461 | 153,7 | 101.3 |
| *Cephalopholis fulva* | Coney | 2 | 350 | 175 | 77.8 |
| *Polydactylus virginicus* | Barbu | 5 | 337 | 67,4 | 27.4 |
| *Holacanthus ciliaris* | Queen angelfish | 1 | 330 |  |  |
| *Thalassophryne nattereri* | Copper Joe | 2 | 279 | 139,5 | 13.4 |
| *Alectis ciliaris* | African pompano | 3 | 257 | 85,7 | 40.1 |
| *Syacium micrurum* | Channel flounder | 2 | 218 | 109 | 1.4 |
| *Pseudupeneus maculatus* | Spotted goatfish | 2 | 205 | 102,5 | 24.7 |
| *Haemulon melanurum* | Cottonwick grunt | 1 | 200 |  |  |
| *Syacium papillosum* | Dusky flounder | 2 | 192 | 96 | 22.6 |
| *Diodon sp* |  | 1 | 170 |  |  |
| *Diapterus auratus* | Broad shad | 1 | 149 |  |  |
| *Haemulon squamipina* | Haemulon squamipinna | 1 | 86 |  |  |
| *Selene setapinnis* | Atlantic moonfish | 1 | 71 |  |  |
| *Harengula clupeola* | False herring | 1 | 45 |  |  |
